# Pulmonary vascular inflammation with fatal coronavirus disease 2019 (COVID-19): possible role for the NLRP3 inflammasome

**DOI:** 10.1186/s12931-022-01944-8

**Published:** 2022-02-10

**Authors:** Oindrila Paul, Jian Qin Tao, Eric West, Leslie Litzky, Michael Feldman, Kathleen Montone, Chamith Rajapakse, Christian Bermudez, Shampa Chatterjee

**Affiliations:** 1 Institute for Environmental Medicine and Department of Physiology, University of Pennsylvania Perelman School of Medicine, 3620 Hamilton Walk, Philadelphia, PA 19104 USA; 2 Department of Pathology, University of Pennsylvania School of Medicine, Philadelphia, PA 19104 USA; 3 Department of Radiology, University of Pennsylvania School of Medicine, Philadelphia, PA 19104 USA; 4 Department of Surgery, University of Pennsylvania School of Medicine, Philadelphia, PA 19104 USA

**Keywords:** NLRP3 inflammasome, Lung inflammation, Microthrombosis, COVID-19, Vascular endothelium, Mechanical ventilation

## Abstract

**Background:**

Pulmonary hyperinflammation is a key event with SARS-CoV-2 infection. Acute respiratory distress syndrome (ARDS) that often accompanies COVID-19 appears to have worse outcomes than ARDS from other causes. To date, numerous lung histological studies in cases of COVID-19 have shown extensive inflammation and injury, but the extent to which these are a COVID-19 specific, or are an ARDS and/or mechanical ventilation (MV) related phenomenon is not clear. Furthermore, while lung hyperinflammation with ARDS (COVID-19 or from other causes) has been well studied, there is scarce documentation of vascular inflammation in COVID-19 lungs.

**Methods:**

Lung sections from 8 COVID-19 affected and 11 non-COVID-19 subjects, of which 8 were acute respiratory disease syndrome (ARDS) affected (non-COVID-19 ARDS) and 3 were from subjects with non-respiratory diseases (non-COVID-19 non-ARDS) were H&E stained to ascertain histopathological features. Inflammation along the vessel wall was also monitored by expression of NLRP3 and caspase 1.

**Results:**

In lungs from COVID-19 affected subjects, vascular changes in the form of microthrombi in small vessels, arterial thrombosis, and organization were extensive as compared to lungs from non-COVID-19 (i.e., non-COVID-19 ARDS and non-COVID-19 non-ARDS) affected subjects. The expression of NLRP3 pathway components was higher in lungs from COVID-19 ARDS subjects as compared to non-COVID-19 non-ARDS cases. No differences were observed between COVID-19 ARDS and non-COVID-19 ARDS lungs.

**Conclusion:**

Vascular changes as well as NLRP3 inflammasome pathway activation were not different between COVID-19 and non-COVID-19 ARDS suggesting that these responses are not a COVID-19 specific phenomenon and are possibly more related to respiratory distress and associated strategies (such as MV) for treatment.

**Supplementary Information:**

The online version contains supplementary material available at 10.1186/s12931-022-01944-8.

## Introduction

It has been more than two years since the pandemic caused by the novel SARS-CoV-2 corona virus (severe acute respiratory syndrome coronavirus), also known as COVID-19 has affected large populations globally [1, 2]. The virus disproportionately affects the respiratory system and a major cause of fatality is the acute respiratory distress syndrome (ARDS) that accompanies the infection [3, 4]. Autopsy-based lung histological studies have been an invaluable tool in understanding the pathobiology of COVID-19; indeed these have shown indications of inflammation, edema, coagulopathy and fibrosis [3, 5–8].

In the pulmonary system, COVID-19 may progress to acute respiratory distress syndrome (ARDS) [9, 10]; incidence of ARDS with COVID-19 is about 33%. However, a mechanistic understanding of this progression remains unclear. Histological, biochemical and physiological studies of COVID-19 have shown extensive inflammation and injury, but the extent to which these are a COVID-19 specific, or an ARDS related phenomenon is not clear. Post mortem findings in COVID-19 affected have shown alveolar damage, early or intermediate proliferative phase, and presence of thrombi and signs of inflammation in the lungs [3, 6, 8] all of which are features common to ARDS from other causes [11–13]. Furthermore, while lung hyperinflammation with ARDS (whether from COVID-19 or from other causes) has been well studied, there is scarce documentation of vascular inflammation in COVID-19 affected lungs [7, 14].

The vascular endothelium, a dynamically adaptable interface that is actively involved in recruitment of inflammatory cells, is well accepted to play a crucial role in regulation, progression, and amplification of inflammation. Inflammatory processes involve the participation of inflammasomes that are multimeric platforms assembled in response to pathogenic stimuli. Dysregulated inflammasome signaling has been well established as a pivotal event in hyper-inflammatory syndromes [15–17]. Among the inflammasomes, the NLRP3 inflammasome comprising of the NLRP3 subunit, ASC and caspase 1, is well established to be activated in response to microbial infection [18, 19], mechanical ventilation (MV, associated with ARDS management) [20] and to drive cell death [21, 22]. It is also involved in ARDS (with or without COVID-19), as evidenced by the detection of inflammasome subunits and products in the sera and lung tissue of ARDS [23, 24] and COVID-19 patients [25, 26].

The purpose of this study is to contextualize vascular features and NLRP3 expression along the vascular wall in lungs of fatal cases of “COVID-19 ARDS” and “non-COVID-19 ARDS” (as compared to lungs from non-COVID-19 non-ARDS subjects) to ascertain if the NLRP3 inflammasome pathway is COVID-19 (SARS-CoV-2 infection) specific or arises from respiratory distress (and associated clinical maneuvers such as MV). Here we document the major histological findings of 8 postmortem examinations done on patients with clinically confirmed COVID-19 and compare these to lungs of 11 non-COVID-19 subjects. This study contributes to the growing data on this topic [3, 6, 26–30].

## Materials and methods

We analyzed lung tissue samples of 8 patients that died of COVID-19 ARDS and 11 patients that died from non-COVID-19 complications in 2020. Of these 11, 8 were non-COVID-19 ARDS while 3 were non-COVID-19 non-ARDS.

Written informed consent was obtained for postmortem examination from the next of kin of these patients. For the COVID-19 patients, SARS-CoV-2 infection was confirmed by real time PCR analysis either at the time of hospital admission or elsewhere (as in the case of Patient 1). All patients except patients 1 and 8 were tested with the Cepheid Gene Xpert RT PCR assay (Cepheid, Sunnyvale, CA 94089). Normal reference range is not detected. The test done on Patient 1 was unknown. Patient 8 had been hospitalized at an outside hospital with confirmed COVID-19 infection and returned to the Hospital of the University of Pennsylvania at the time of readmission. Autopsies were done by trained personnel using personal protective equipment in accordance with the recommendations of the University of Pennsylvania School of Medicine.

Multiple random sampling of postmortem lung tissue was used so as to adequately represent each lung. Tissues were fixed in formalin. Paraffin embedded sections of 3 to 5 μm thickness were stained with hematoxylin and eosin (H&E). Images were captured on the Aperio Pathology System and visualized by ImageScope (Leica Biosystems, Buffalo Grove, IL). High (× 20) and low powered fields (× 1) were selected for evaluation. 3–4 fields were analyzed for each subject. Imaging and scoring were done by different observers (blinded). Scores were given based on image assessment by computationally deriving 10 sections (for each field) that were assessed at × 20. Hyaline membranes, interstitial fibrosis, atypical pneumocytes and pulmonary hemorrhage were assessed by a scoring system that depended upon the % of image area involved as described by us earlier [31–33]. A scale of 0–4 was used: 0 is absent; 1 = mild or 25% of the area; 2 = moderate or 50% of the area; 3 = high or 75%; 4 = severe or 90–100% of the image area. Scores provided are average of 3 fields for each subject.

Additional sections from the same samples were employed to assess inflammation and inflammation induced cell death (pyroptosis) by immunostaining for NLRP3 inflammasome and caspase 1 respectively. Double labeling of sections was also performed to immunostain for both NLRP3 and caspase 1. Sections were deparaffinized and hydrated followed byantibody retrieval and immunostainingby using anti-human NLRP3 monoclonal antibody at 1:200 or anti-human caspase antibody at 1:100 (both from R&D Systems, Minneapolis, MN). Secondary antibody used was conjugated to Alexa 488 at 1:200 (Life Technologies, Eugene, OR). Appropriate IgG controls were used to fix exposure settings (Additional file 1: Fig. S1). Vectashield antifade mounting medium used was from Vector Labs (Burlingame, CA). Images were acquired by using a Nikon TMD epifluorescence microscope equipped a Hamamatsu ORCA-100 digital camera, and MetaMorph imaging software (Universal Imaging, West Chester, PA, USA). For double labeling of NLRP3 and caspase 1, monoclonal NLRP3 and polyclonal caspase 1 antibodies were used. Leica TCS SP8 confocal microscope with super-resolution imaging of green and red dyes (with adjusted laser power and for the individual detection channels to avoid bleed through) was used. Fluorescence images were acquired at λ_excitation_ = 488 nm for green and λ_excitation_ = 595 nm for red; all images were acquired with the same exposure and acquisition settings as reported previously [34–36]. Quantitation of the fluorescence signal was carried out using the MetaMorph Imaging Software. Integrated Intensities were normalized to the field area as reported by us elsewhere [34]. Results are presented as mean ± standard deviation (SD). Group differences were evaluated by ANOVA followed by Tukey post hoc comparisons. Statistical significance for all studies was accepted as p < 0.05.

## Results

Patient demographics and clinical information are summarized in Table 1, and histological characteristics in Table 2. COVID-19 patients were 4 men and 4 women, with a mean age of 71.8 years (SD 13.9); non-COVID-19 patients comprising of non-COVID-19 ARDS and non-COVID-19 non-ARDS were 7 men and 4 women, with a mean age of 64 (SD 10.7). Lung sections from all COVID-19 ARDS and non-COVID ARDS patients showed diffuse alveolar damage including hyaline membranes, intra-alveolar fibrin deposition, and thickening of the alveolar-capillary membrane. All sections from lungs of COVID-19 ARDS, non-COVID-19 ARDS subjects stained positively for the NLRP3 inflammasome associated markers that were assessed by fluorescence imaging.

**Table 1:** Patient numbers 1–8 were COVID-19 positive

Patient	Gender	Age	Race	Known medical history	Substance abuse (smoking/alcohol)
1	Female	61	W	Asthma and stroke	Non- smoking
2	Female	63	W	Breast cancer and therapy related acute leukemia	Smoking
3	Female	73	W	COPD	Smoking and Alcohol
4	Female	94	W	COPD, Coronary Artery Disease and Siogrens disease	Not known
5	Male	50	B	Myeloproliferative disorder and Pulmonary/portal hypertension	Not known
6	Male	72	B	Dementia, diabetes and hypertension	Not known
7	Male	74	B	Pulmonary Embolsim and deep vein thrombosis and hypertension	Not known
8	Male	85	U	Cerebral Vascular disease	Not known
9	Male	63	W	Heart Transplant	Not known
10.*	Female	59	B	Emphysema	Not known
11.*	Male	81	B	Emphysema	Not known
12.*	Male	40	B	Bronchopneumonia	Not known
13.*	Female	62	W	Diffuse alveolar damage; chronic lung disease	Not known
14.*	Male	72	B	End stage lung disease	Not known
15.^*^	Female	68	W	Diffuse alveolar damage; COPD and renal cell carcinoma	Not known
16	Female	69	B	Breast Cancer, Mild edema in lung	Not known
17.^*^	Male	77	U	Diffuse alveolar damage and COPD	Not known
18.*	Male	73	B	Aspiration pneumonia; diabetes	Not known
19	Male	57	W	Sarcoid	Not known

Patient numbers 9–19 were non-COVID-19. Of these ^*^ denote non COVID-19 ARDS. The rest were (Patient numbers 9, 16, 19) were non-COVID-19 non-ARDS. B, W and U denote black, white and unknown respectively

**Table 2:** Pulmonary pathological features from autopsy cases of COVID-19 ARDS (patient no 1–8), non-COVID-19 ARDS (10–15, 17–19) and non-COVID-19 non-ARDS (9, 16, 19)

Patient No	Hyaline Membranes	Interstitial Fibrosis	Atypical pneumocytes	Pulmonary hemorrhage	Trombi/Microthrombi	Morphological aspects
1	4	4	4	3	2	Proliferative phase of diffuse alveolar damage, thrombi/microthrombi
2	3	3	4	3	3	Emphysematous change, microthrombi, alveolar septal thickening, thrombi/microthrombi
3	4	4	4	3	3	Pulmonary edema, alveolar septal thickening
4	4	4	4	4	3	Proliferative phase of diffuse alveolar damage, pulmonary hemorrhage, thrombi/microthrombi
5	4	4	4	3	3	Diffuse alveolar damage, Advanced proliferative phase, thrombi/microthrombi
6	4	4	4	4	3	Advanced proliferative phase, pulmonary hemorrhage, thrombi/microthrombi
7	4	4	4	4	3	Exudative phase diffuse alveolar damage, hemorrhage, thrombi/microthrombi
8	4	4	4	4	3	Advanced proliferative phase, hemorrhage, thrombi/microthrombi
9	2	3	2	2	1	Edema
10	3	3	4	3	1	Diffuse alveolar damage, hemorrhage in some regions
11	4	4	4	1	1	Edema, hemorrhage, microthrombi, features of fibroblastic phase
12	4	4	4	3	2	Edema, exudates in lung parenchyma, fibrotic regions, interstitial inflammation and microthrombi
13	4	4	4	4	1	Diffuse alveolar damage Pulmonary edema, alveolar septal thickening
14	4	4	4	3	1	Epithelial cell denudation, alveolar collapse
15	4	4	4	2	2	Edema, exudates in lung parenchyma, hemorrhage, thrombi/microthrombi
16	1	1	1	1	1	Minor edema in some alveoli
17	3	2	2	3	2	Fibroblast proliferation, microthrombi
18	4	4	4	4	2	interstitial and intra-alveolar fibroblast proliferation, microthrombi
19	2	2	2	1	1	Edema

A scale of 0–4 was used for each subject: 0 is absent; 1 = mild or 25% of the field area; 2 = moderate or 50% of the field area; 3 = high or 75%; 4 = severe or 90–100% of the image area. Scores provided are mean of 3–4 fields for each subject

**Fig. 1:**
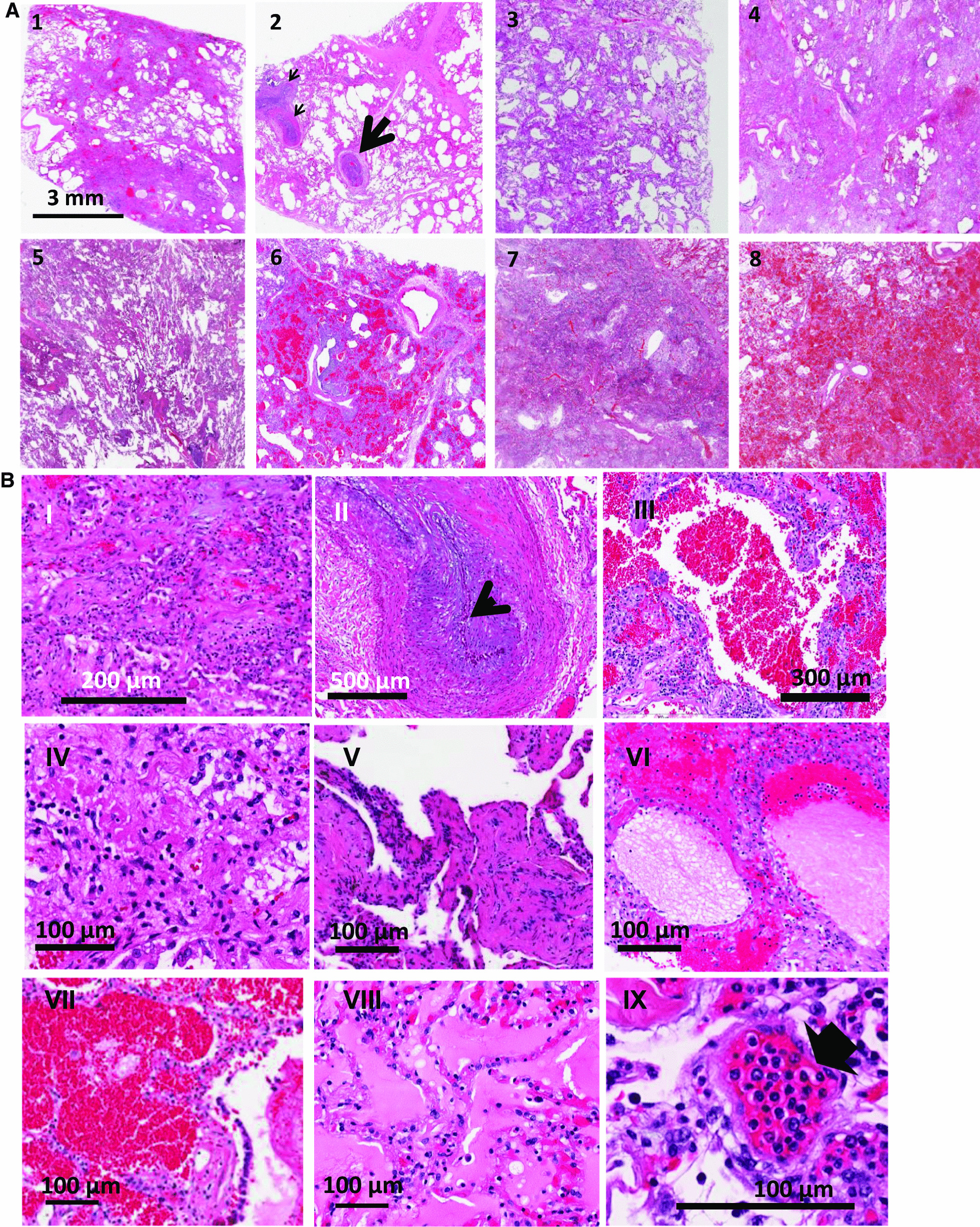

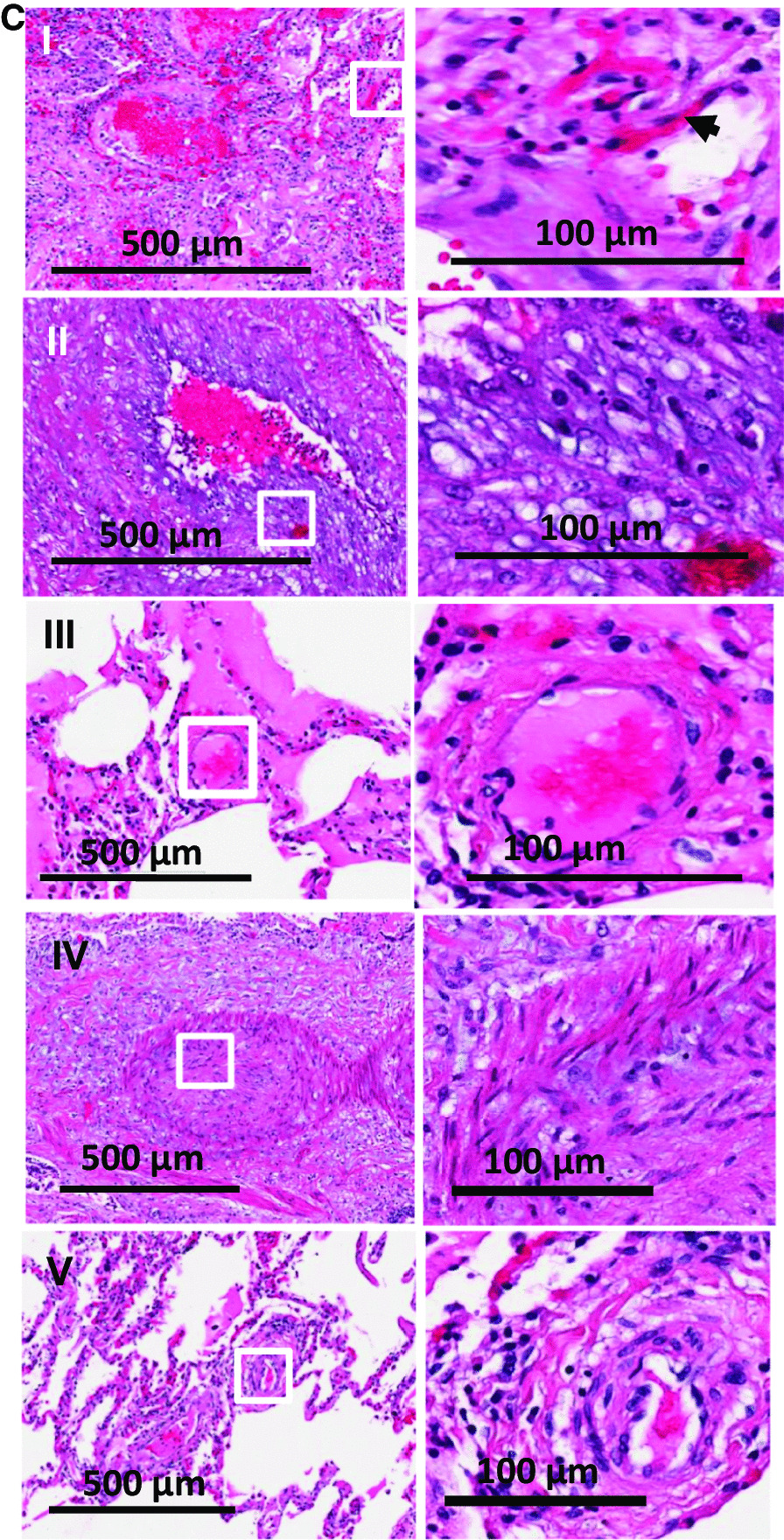
Hematoxylin and eosin-stained sections staining from representative regions of the lung parenchyma of post-mortem lung tissue of 8 COVID-19 patients (Patient 1 to 8). **A** All patients show extensive alteration of lung microstructure in the form of alveolar damage, fibrin exudation into alveolar space, thrombi and fibroblastic proliferation. The septa are thickened and there is presence of hyaline membranes and dense infiltrates. Scale bar is 3 mm. 1: patient 1- Alveolar damage with collagen deposition and exudative pattern of damage, 2. patient 2- Large thrombi and smaller caliber arteries showing fibrin thrombi (arrows), 3. patient 3-Alveolar damage pattern arising from fibroblastic proliferations, 4 and 5. patient 4 and 5- Exudate in the entire lung, 6. patient 6-Necrosis with blood and exudate in the lung parenchyma, 7. patient 7-Hemorrhagic infarction of lung tissue adjacent to a pulmonary artery with thrombotic material, 8. patient 8-Pulmonary hemorrhage with blood and fibrin exudation into the parenchyma. **B** H and E staining at higher magnification to show representative areas of extensive diffuse alveolar damage, microthrombi and edema in regions of the lungs from various COVID-19 ARDS samples. I. Fibroblastic proliferation, II. Plugged airway due to remodeling, III. Coagulation necrosis with blood in the lung tissue, IV. Proliferative phase of diffuse alveolar damage, V. Patchy distribution of damage, VI. Proteinaceous exudates in alveolar spaces, VII. Blood and fibrin exudation into parenchyma, VIII. Proteinaceous exudates in alveolar spaces, IX. Endotheliitis of small vessel < 100 μm with infiltration of the vessel wall by lymphocytes (arrow shows infiltrated cells). **C** Thrombi and microthrombi were identified in lung sections of 7 of the 8 patients. Images of vessels were chosen to emphasize the microthrombi. Box is magnified in the right panel. Arrow shows microthrombi on alveolar septa

Upon light microscopic examination, the lungs of all COVID-19 ARDS patients showed extensive alteration of lung microstructure (Fig. 1A, B). A closer inspection of COVID-19 lungs revealed fibrin exudation into alveolar space, extensive thrombi and fibroblastic proliferation, hyaline membrane, fibrin deposition and early and advanced proliferative phase of diffuse alveolar damage (Fig. 1B). Thrombi and microthrombi were identified in sections from 7 of the 8 patients (Fig. 1C). Vascular changes were extensive, with microthrombi in small vessels and arterial thrombosis and organization. Microthrombi were also observed in alveolar septa. Thrombi and microthrombi were found in > 75–80% of the fields imaged. Histological findings are detailed in the legends of Fig. 1 and in Table 2.

**Fig. 2:**
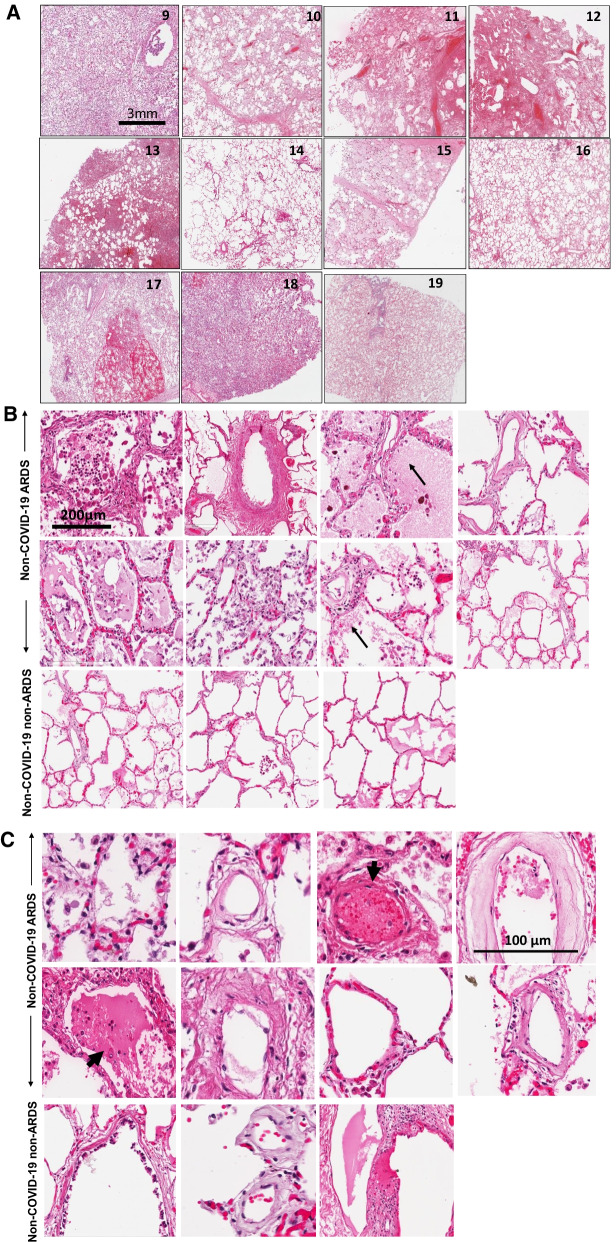
**A**. Hematoxylin and Eosin-stained sections staining from representative regions of the lung parenchyma of post-mortem lung tissue of 8 non-COVID-19 ARDS (patient numbers 10–15, 17,18) and 3 non-COVID-19 non-ARDS (patient numbers 9,16,19) patients. Scale bar is 3 mm. **B** H and E staining at higher magnification: Diffuse alveolar damage, microthrombi and edema were observed. Arrows show proteinaceous exudate in the airspaces. Scale bar is 200 microns**. C** Vascular structures in lungs from non-COVID-19 sources. Arrows show thrombi in vessels. About 40% of the fields from non-COVID ARDS showed thrombi. Very few microthrombi were observed in non-COVID non-ARDS lungs. Scale bar is 100 microns

In contrast, the lungs from non-COVID ARDS, showed less thrombi and fibrin exudation (Fig. 2A, B). While higher magnification showed certain key features of lung injury such as diffuse alveolar damage, thickening of the alveolar-capillary membrane, fibroblastic proliferation, the presence of hyaline membranes, edema and proliferative phase of diffuse alveolar damage, there was overall an intact structure and less alveolar infiltration or hemorrhage (Fig. 2 B). Thrombi were present in < 40% of the fields (Fig. 2C). In terms of structure, the non-COVID-19 non-ARDS lungs (9, 16 and 19, see Table 1) were relatively intact and showed almost no thrombi (Fig. 2B and C, lower panel). Histological findings are detailed in Table 2.

**Fig. 3:**
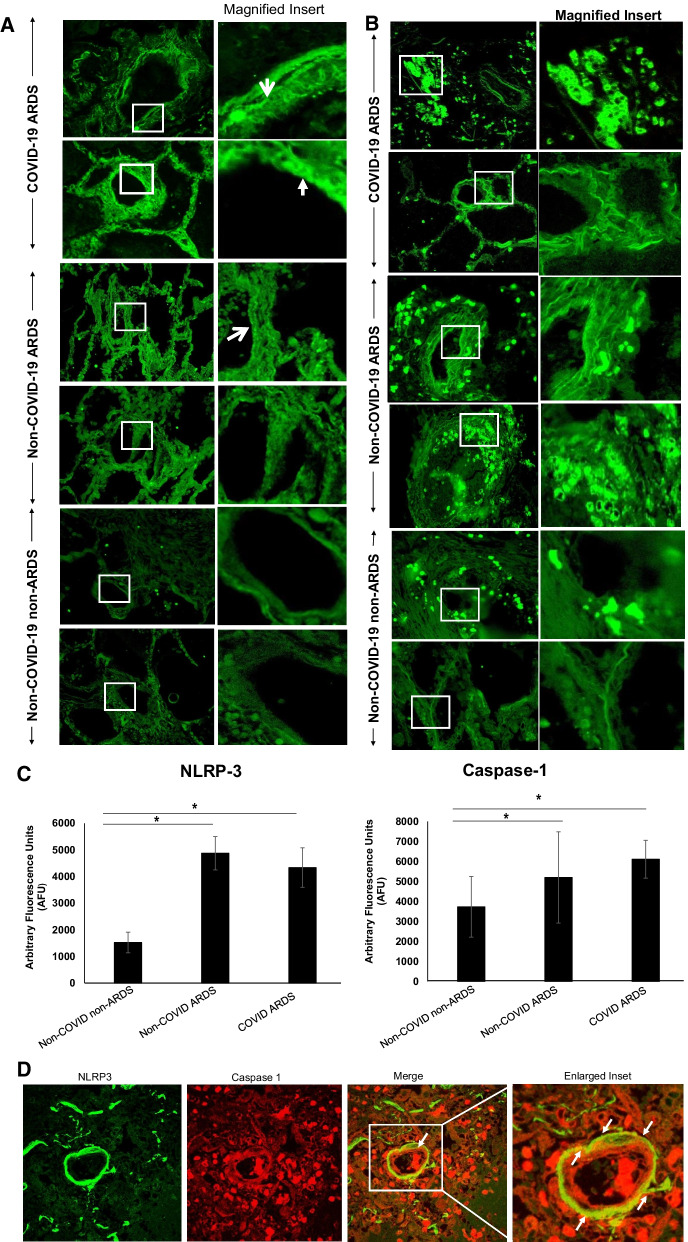
Inflammasome in the lungs of patients with COVID-19 ARDS, non-COVID-19 ARDS and non-COVID-19 non-ARDS. Representative images of the immunofluorescence in lung sections stained with anti-NLRP3 and Caspase 1. **A** The NLRP3 subunit (green) along the walls of arterioles (arrow). Upper panels: COVID-19 ARDS lungs. Middle Panels: Lungs from non-COVID-19 ARDS subjects. Lower Panels: Lungs from non-COVID-19 non-ARDS subjects, without respiratory disease. **B** Caspase staining (green). Upper panels: Upper panels: COVID-19 ARDS lungs. Middle Panels: Lungs from non-COVID-19 ARDS subjects. Lower Panels: Lungs from non-COVID 19 non-ARDS subjects, without respiratory disease. **C** Quantitation of the fluorescence intensity of the images using MetaMorph Imaging Program. *p < 0.01 as compared to non-COVID non-ARDS lungs. Results are presented as mean ± standard deviation (SD). Group differences were evaluated by ANOVA followed by Tukey post hoc comparisons. Statistical significance was accepted as p < 0.05. **D** NLRP3 (green) and caspase 1 (red) in lung sections of patients with COVID-19. Arrows show colocalization (yellow) in regions along the vascular wall

We next assessed the expression of the NLRP3 subunit and its downstream effector caspase 1 in all samples. In lungs from COVID-19 ARDS and non-COVID-19 ARDS subjects, intense expression of the NLRP3 and caspase 1 was observed as visualized from the green-fluorescent signal, as shown in Fig. 3A and B (two upper and middle panels). For COVID-19 affected subjects, fluorescence around the vessel walls implied NLRP3 expression along the endothelial layer (Fig. 3A, two upper panels). The effector enzyme, caspase 1 was widely distributed throughout the lungs and was not limited to the vascular structures (Fig. 3B, two upper panels). Non-COVID-19 ARDS lungs showed a similar staining pattern (Fig. 3A and B, two middle panels). Indeed, NLRP3 and caspase 1 expression were not significantly different from COVID-19 lungs (Fig. 3C). Non-COVID non-ARDS lungs (Patient 9,16 and 19, see Table 1) showed significantly lower NLRP3 and caspase 1 expressions as compared to the two other cohorts (COVID-19 ARDS and non-COVID-19 ARDS) (Figs. 3A, B lower two panels and Fig. 3C). Lungs from COVID-19 ARDS subjects co-labeled for NLRP3 and caspase 1, showed colocalization (yellow) along the vessel wall. In these lungs, caspase 1 expression was high throughout the tissue, but NLRP3 expression was visualized along the vascular structures (Fig. 3D).

## Discussion

COVID-19 has been described largely as a respiratory disease; indeed, the respiratory tract and alveoli are amongst the primary sites of infection. However, it is also an inflammatory disease where release of inflammatory cytokines is the cause of organ injury and damage. The endothelium is the converging site of the inflammation as its activation (expression of adhesion molecules and cytokines) leads to immune cell recruitment; thus, it is reasonable to conclude that COVID-19 is potentially a vascular disease that has its origins in “endothelial inflammation” signaling [14, 37, 38].

Our inspection of lung autopsies of the 8 COVID-19 patients showed macro and microthrombi in almost all fields imaged (7 out of 8 subjects showed extensive microthrombi/thrombi in all lung sections), indicating coagulation pathology. For non-COVID-19 ARDS lungs, about 40% of the sections showed microthrombi. Very few thrombi were observed in the sections of non-COVID-19 non-ARDS lungs. As coagulation is closely linked to endothelial inflammation signaling, the presence of pulmonary thrombi with COVID-19, as observed here and reported elsewhere [39], indicates endothelial inflammation which can increases leukocyte infiltration and alter coagulation control driving a procoagulant direction [40].

A pivotal molecule that drives endothelial inflammation and injury is the NLRP3 inflammasome. It is a multiprotein complex comprised of three basic components: (1) A sensor such as a NOD-like receptor (NLR) (2) the adaptor protein apoptosis-associated speck-like protein containing a caspase-recruitment domain (ASC) and (3) the inflammatory cysteine aspartase caspase 1. The assembly of this complex leads to release of caspase 1 which then exerts its catalytic activity on the pro-inflammatory cytokine (IL-1β) that after their release perpetuate cell death, specifically inflammation induced cell death or pyroptosis [21, 22].

While a recent report showed high levels of NLRP3 inflammasome and caspase 1 expression in lungs with fatal COVID [26], the status of the NLRP3 inflammasome on the pulmonary vascular wall in COVID-19 is not known. Our data showed expression of NLRP3 and caspase 1 along the vascular wall with COVID-19 ARDS and non-COVID-19 ARDS (Fig. 3A–C). COVID-19 ARDS and non-COVID-19 ARDS samples showed comparable expressions of NLRP3 and caspase 1. We also observed that while both NLRP3 and caspase-1 were highly expressed along the vascular wall in COVID-19 ARDS samples, there was extensive colocalization of these two moieties. (Fig. 3D). As caspase 1 is the downstream effector of NLRP3, colocalization indicates activation of the NLRP3 inflammasome in the vessel wall. Caspase 1 can potentially drive endothelial cell injury via pyroptosis. This confluence of vascular injury, thrombosis and dysregulated inflammation seems to support a pivotal role for the pulmonary endothelium in severe and fatal COVID-19.

Increased NLRP3 is, of course, associated with various inflammatory lung pathologies and aberrant activation of NLRP3 inflammasome contributes to ARDS induced lung inflammation and injury [23, 24, 41]. This is because chemotactic signals associated with microbial attack and/or stretch signaling associated with MV (a standard therapy to maintain adequate gas exchange during ARDS) activate the NLRP3 inflammasome in alveolar macrophages [20, 42]. Caspase 1 which has been reported to show an increase with ARDS [43] and MV [44] and has recently been found to increase with COVID-19 [26]. Thus, the fact that both the COVID-19 ARDS and non-COVID-19 ARDS lung autopsies in this study showed high NLRP3 and caspase 1 expression (as compared to non-COVID-19 non-ARDS lungs) seems to suggest that the activation of NLRP3 inflammasome pathway is more related to ARDS (and associated MV) and may not be a COVID-19 related phenomenon alone.

**Fig. 4:**
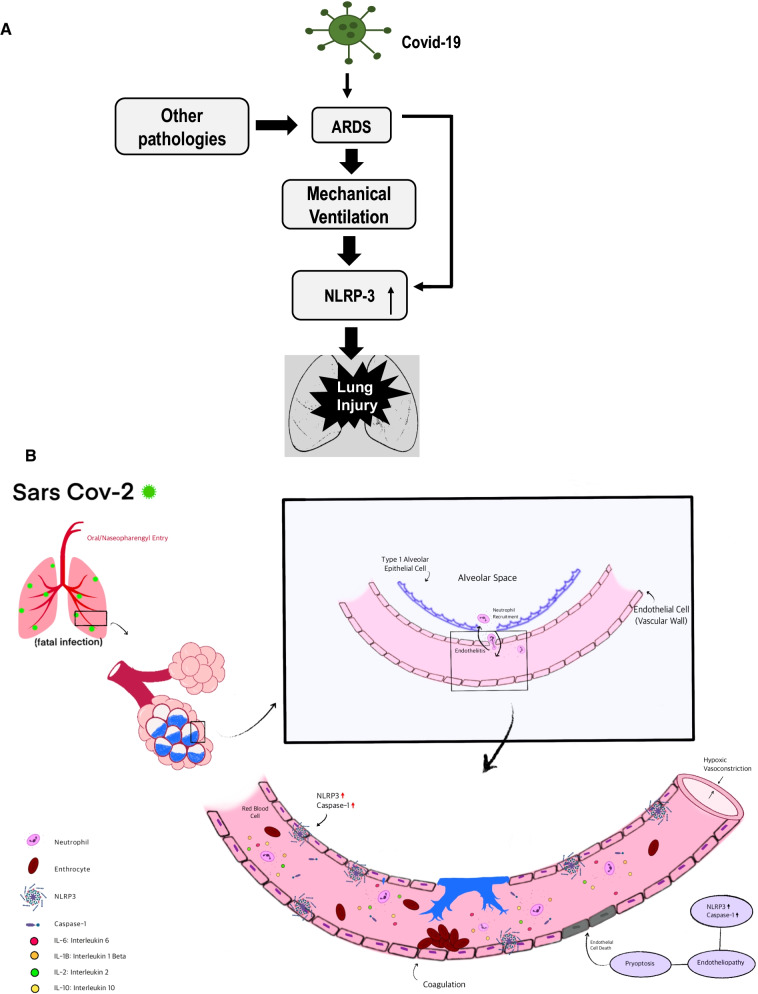
**A** ARDS (and mechanical ventilation) associated with COVID-19 seems to be largely responsible for activation of the NLRP3 inflammasome. This pathway seems to be ARDS related and not COVID-19 (SARS CoV2 virus infection) specific. **B** Overview of SARS-CoV-2 entry, infection and endothelial inflammation and cell death. As is well established, oral nasopharyngeal entry of SARS-CoV-2 is followed by its binding to the alveolar epithelium. The infected pneumocytes secrete cytokine and chemokines, which attract neutrophils to the alveolar space, leading to a possible breach of the alveolar wall. Meanwhile, endothelial cells overexpress NLRP3 as we observed in the autopsies (either by infection, or via increased amounts of chemokines and cytokines). The NLRP3 pathway drives endothelial pyroptosis. This leads to breakdown of the endothelial-alveolar barrier and causes interstitial and alveolar space flooding. Endothelial cell death and debris activate coagulation cascades that promotes thrombi formation

There is some speculation on the mechanisms by which inflammasome activation occurs upon SARS-CoV-2 infection. There are several possibilities of NLRP3 activation with COVID-19. One possibility is the binding of SARS-CoV-2 spike protein to angiotensin-converting enzyme 2 (ACE2) directly and subsequent activation of NLPR3 inflammasome via altered membrane polarity [45]. Another possibility could be via interaction of damage associated molecular patterns (DAMPs that are released post microbial infection and MV [20, 46, 47] and members of the complement cascade with the SARS-CoV-2 virus [48]. However, from our data it seems likely that it is ARDS and MV (stretch from MV is known to activate the inflammasome [42]) associated with COVID-19 that drives the NLRP3 pathway (Fig. 4A). Once activated around the vascular wall (endothelial layer), the NLRP3 inflammasome would lead to release of caspase 1 and interleukin-1β that would facilitate pyroptosis (cell death) of the endothelium (Fig. 4B).

To the best of our knowledge, this is the first study on NLRP3 expression in the vascular structures in lungs of fatal cases of COVID-19. The origin of several events that exacerbate inflammation and injury with COVID-19 (such as immune cell aggregation and extravasation, edema, formation of thrombi and leukopenia) possibly lies in pulmonary endothelial inflammasome activation and pyroptotic cell death. Therefore, NLRP3 inhibitors have been suggested for as a potential treatment strategy and are currently being explored for management of moderate COVID-19 symptoms (NCT04540120).

A major drawback of this study is the low statistical power owing to the small sample size. Moreover, paraffin based post-mortem samples offer a snapshot of the disease and cannot recreate the evolving disease process. Histology is also impacted with the effects of clinical care and treatment as comorbidities, ventilation and medication pose as challenges in interpretation of results. Nevertheless, despite these caveats this study identifies vascular endothelial NLRP3 inflammation, and documents thrombi and altered vascular structures in the lungs of fatal COVID-19 patients.

## Conclusions

Taken together, our data show that NLRP3 inflammasome pathway activation was not different between COVID-19 and non-COVID-19 ARDS suggesting that this pathway is not COVID-19 specific and is possibly more related to respiratory distress. However, the fact that there is a role for NLRP3 inflammasome pathway with SARS-CoV-2 infection indicates that a potential usage of antagonists or blockers of the NLRP3 pathway in COVID-19 inflammation regulation and control. Overall, this report adds to the growing list of studies on COVID-19 associated pulmonary pathology that highlight the importance of vascular endothelial inflammation in progression to severe and fatal disease.

## Supplementary Information

**Additional file1: Figure S1.** To control for signal arising from IgG immunoreactivity, IgG isotype controls were used: A. Rat IgG isotype controls, B. Nuclear stain DAPI to show cells in the same section, C. Goat IgG isotype controls and D. DAPI.

## Data Availability

The samples, datasets, imaging parameters and analysis of this study are available from the corresponding author on reasonable request**.**

## Abbreviations

ACE2: Angiotensin-converting enzyme 2
ARDS: Acute respiratory distress syndrome
ASC: Apoptosis-associated speck-like protein containing a caspase-recruitment domain
COVID-19: Coronavirus disease of 2019.
H&E: Hematoxylin and eosin
MV: Mechanical ventilation
NLRP3: NOD-like receptor protein 3
SARS-CoV-2: Severe acute respiratory syndrome coronavirus
