# Supplementary material for: Pulmonary vascular inflammation with fatal coronavirus disease 2019 (COVID-19): possible role for the NLRP3 inflammasome
Source: Respir Res. 2022 Feb 10;23:25. doi: 10.1186/s12931-022-01944-8 (PMC8830114; doi:10.1186/s12931-022-01944-8)
Supplement: Supplementary file 1 — Additional file1: Figure S1. To control for signal arising from IgG immunoreactivity, IgG isotype controls were used: A. Rat IgG isotype controls, B. Nuclear stain DAPI to show cells in the same section, C. Goat IgG isotype controls and D. DAPI. [file 12931_2022_1944_MOESM1_ESM.pdf]

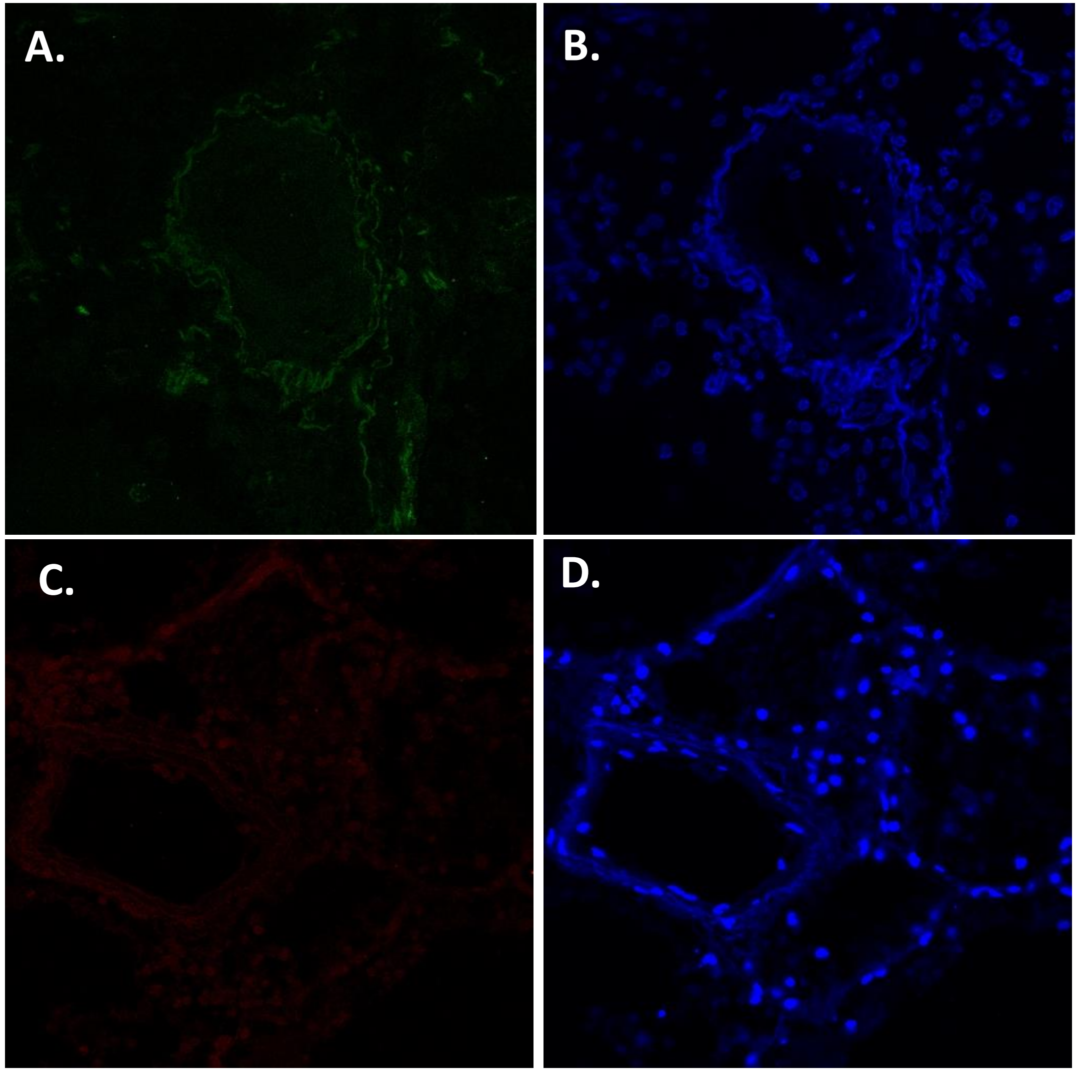

Supplemental Figure S1: To control for signal arising from IgG immunoreactivity, IgG isotype controls were used:

A. Rat IgG isotype controls, B. Nuclear stain DAPI to show cells in the same section

C. Goat IgG isotype controls and D. DAPI.
